# Cortical Hierarchical Disorganization and Transcriptomic Associations in Multiple Sclerosis and Neuromyelitis Optica Spectrum Disorders Revealed by Morphometric INverse Divergence Network Gradients

**DOI:** 10.1002/cns.71240

**Published:** 2026-11-09

**Authors:** Yingying Xie, Shiqi Lin, Jie Sun, Han Zhang, Jiaojiao Du, Zhang Zhang, Bohong Zou, Dairong Cao, Ningnannan Zhang

**Affiliations:** ^1^ Department of Radiology The First Affiliated Hospital, Fujian Medical University Fuzhou Fujian China; ^2^ Department of Radiology National Regional Medical Center, Binhai Campus of the First Affiliated Hospital, Fujian Medical University Fuzhou Fujian China; ^3^ Department of Radiology, Tianjin Key Laboratory of Functional Imaging & Tianjin Institute of Radiology Tianjin Medical University General Hospital Tianjin China; ^4^ Department of Breast Imaging Tianjin Medical University Cancer Institute & Hospital Tianjin China; ^5^ Department of Radiology, Fujian Key Laboratory of Precision Medicine for Cancer The First Affiliated Hospital, Fujian Medical University Fuzhou Fujian China; ^6^ Key Laboratory of Radiation Biology of Fujian Higher Education Institutions The First Affiliated Hospital, Fujian Medical University Fuzhou Fujian China

**Keywords:** cortical gradient, morphometric INverse divergence, multiple sclerosis, neuromyelitis optica spectrum disorders, transcriptomic profiling

## Abstract

**Background:**

Multiple sclerosis (MS) and neuromyelitis optica spectrum disorders (NMOSD) are autoimmune neuroinflammatory conditions characterized by widespread brain alterations. However, how cortical hierarchical organization is altered across these disorders and how such alterations relate to underlying molecular architecture remain unclear.

**Methods:**

We applied the Morphometric INverse Divergence (MIND) framework to structural MRI data from 62 MS patients, 53 NMOSD patients, and 74 healthy controls. Cortical gradients were constructed based on MIND features to represent the hierarchical organization across the cortex. The derived MIND‐gradient metrics were integrated with multiscale analyses, including clinical and neuropsychological assessments, neurotransmitter system mapping, and transcriptomic profiling coupled with enrichment analyses, to link macroscale structural organization with molecular and functional substrates.

**Results:**

MIND‐gradient analyses showed alterations in cortical hierarchical organization in MS and NMOSD, mainly involving frontal and parietal regions. The primary MIND gradient was significantly associated with the spatial distributions of multiple neurotransmitter receptors and transporters. Transcriptomic analyses identified 1978 gradient‐associated genes in MS and 2485 in NMOSD. These genes were enriched in immune‐related, synaptic, and transport‐related biological processes in both disorders, with shared and non‐overlapping enriched terms. Cellular enrichment analysis further highlighted enrichment for myelinating oligodendrocytes, and developmental enrichment analysis suggested different temporal enrichment patterns in MS and NMOSD.

**Conclusions:**

These findings provide insights into cortical hierarchical alterations in MS and NMOSD and highlight potential molecular correlates that warrant further investigation in longitudinal studies.

## Graphical abstract

Cortical morphometric gradient analyses suggest regional alterations in multiple sclerosis and neuromyelitis optica spectrum disorder and reveal regional associations with cognitive performance. Integrative transcriptomic analyses suggest shared and divergent molecular signatures, providing insights into the biological context of cortical organization in these disorders.

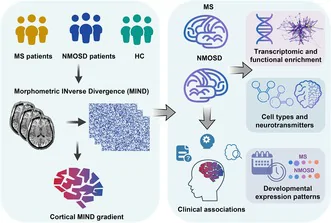

## 1 Introduction

Multiple sclerosis (MS) and neuromyelitis optica spectrum disorders (NMOSD) are autoimmune neuroinflammatory diseases that result in widespread brain structural and functional impairment [1, 2]. Both conditions are characterized by inflammatory demyelination, axonal injury, and neurodegenerative changes, which contribute to substantial neurological disability [3]. Although the two disorders arise from distinct primary pathogenic mechanisms, their clinical manifestations frequently overlap. This phenotypic convergence complicates differential diagnosis and suggests the presence of partially shared, yet differentially organized, neurobiological alterations at the systems level. Although conventional neuroimaging captures focal lesions and atrophy, it often overlooks subtle disruptions in whole‐cortical hierarchical organization that may offer biologically meaningful markers for improving diagnostic precision and understanding clinical and molecular heterogeneity.

Increasing evidence suggests that MS and NMOSD are characterized by disruptions in brain network organization and hierarchical architecture, rather than being solely defined by focal lesions or regional atrophy [4, 5, 6]. In these diseases, inflammatory and neurodegenerative mechanisms drive progressive structural damage, disrupting the organized morphometric and functional architecture of the brain and contributing to accelerated brain aging, cognitive decline, and physical disability. Importantly, such structural damage may interfere with the orderly hierarchical organization of gray matter. Recent advances in network neuroscience have highlighted the importance of cortical gradients, which capture the continuous topographic hierarchy from lower‐level sensory regions to higher‐order association areas [7]. Higher gradient values are typically observed in transmodal regions, representing the integrative end of the cortical hierarchy, whereas lower gradient values in primary sensorimotor regions reflect more specialized unimodal organization [8, 9]. These gradients provide a framework for understanding how brain structure is organized along multiple levels of hierarchy and how disease may perturb these patterns. Besides, the Morphometric INverse Divergence (MIND) framework as an advanced approach of cortical similarity networks analyses [10], has been introduced to analyze the structural damage in MS, bridging the gap between connectomics and clinical practice in MS [11]. Compared with conventional gradient methods that rely on regional averages or coarse parcellations, MIND‐derived gradients may offer enhanced sensitivity to subtle hierarchical disorganization, allowing detection of structural alterations that might be overlooked by traditional approaches. However, direct comparative studies assessing cortical hierarchical disorganization across MS and NMOSD remain limited, leaving a critical gap in our understanding of their shared and specific network‐level alterations.

Beyond structural alterations, growing evidence suggests that neuroinflammatory processes and transcriptomic changes play critical roles in the pathophysiology of MS and NMOSD. In addition, the spatial distribution of cortical gradients was spatially coupled to some microstructural distribution, such as cell‐type, neurotransmitter distribution, and gene expression patterns [9, 12]. Integrating morphometric gradients with these multiscale molecular datasets therefore offers a way to link macroscale cortical metrics and microscale biological processes and to identify the molecular correlates of hierarchical reorganization in demyelinating disease.

In this study, we integrated MIND network gradients with clinical, neurochemical, and transcriptomic analyses to delineate cortical hierarchical organization in MS and NMOSD, using a prospectively collected cohort comprising 62 patients with MS, 53 patients with NMOSD, and 74 healthy controls. Since cortical morphometric hierarchy has not been directly compared between these two disorders, we further examined their shared and disorder‐specific organizational patterns and explored the clinical, neurotransmitter, and transcriptional correlates of hierarchical reorganization. By moving beyond region‐specific morphometric alterations, this approach provides a system framework for understanding convergent and divergent network changes in demyelinating disease. The flowchart of the study design is shown in Figure 1.

**FIGURE 1:**
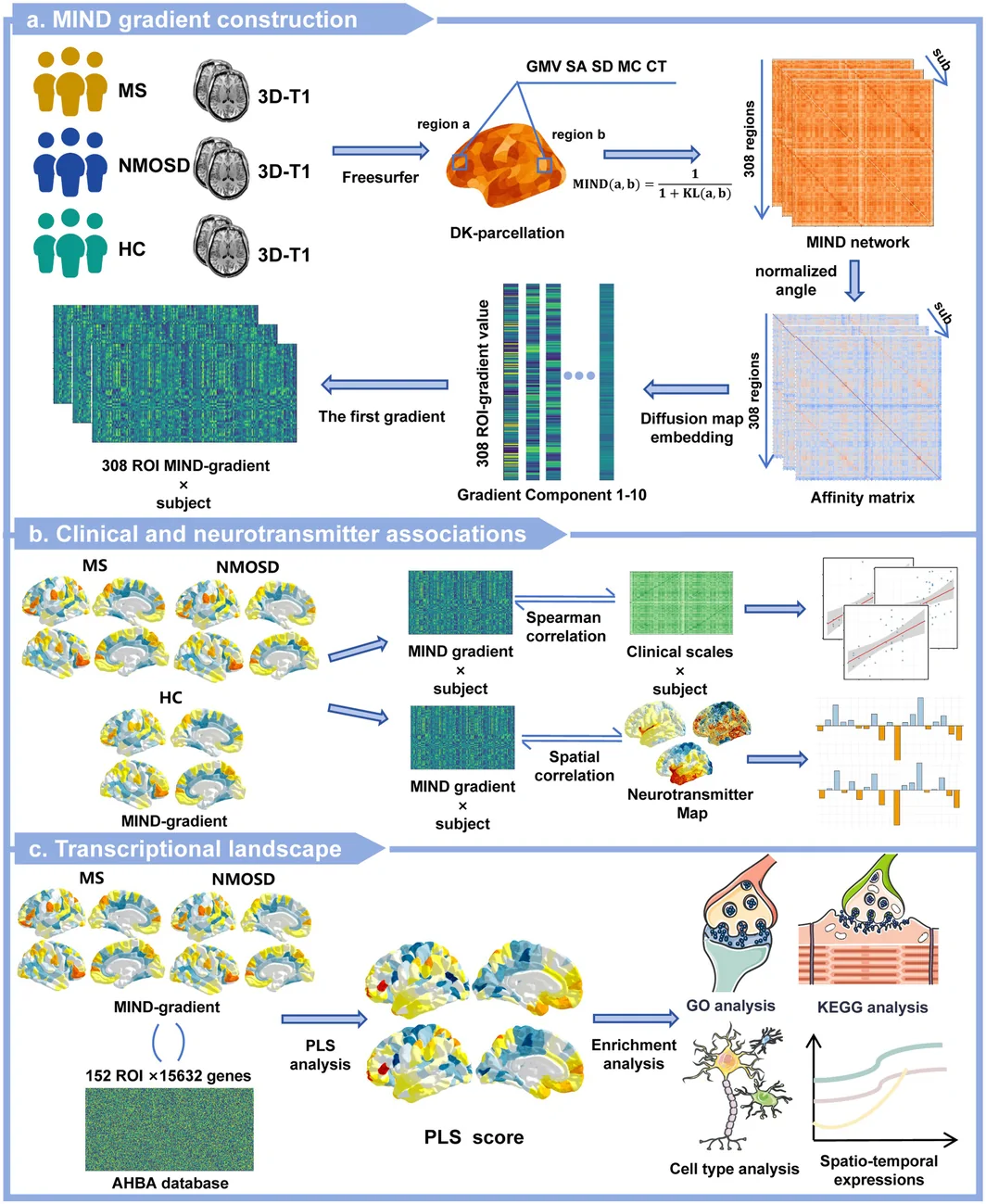
Study overview. (a) Construction of MIND‐gradient. Multidimensional cortical morphological features (GMV, SA, SD, MC, and CT) were used to construct a 308 × 308 morphological similarity matrix for MIND. Then, regional MIND values were obtained by averaging all pairwise connections across the 308 cortical regions, without applying any thresholding. The resulting MIND matrix was converted into an affinity matrix via the normalized angle method. Diffusion map embedding was subsequently performed to derive the MIND network gradients, and analyzed focused on the first gradient, which explained the largest proportion of variance in connectivity. (b) Association of neurotransmitter systems and clinical scales with the primary MIND gradient in MS, NMOSD and HC. The Spearman correlation was used to analyze the relationships between the primary MIND gradient and a series of clinical scales in MS and NMOSD. The spatial relationships between the primary MIND gradient and maps of neurotransmitter receptors or transporters was evaluated. (c) Transcription association analysis. The PLS regression was then employed to identify relationships between gene expression patterns and the primary MIND gradient in MS and NMOSD. To further explore the biological functions of these significant PLS genes, we performed GO analysis (GO‐BP, GO‐CC and GO‐MF), KEGG pathways, cell type analysis and developmental trajectory analysis. CT, cortical thickness; GO‐BP, gene ontology biological processes analysis; GO‐CC, gene ontology cellular component; GO‐MF, gene ontology molecular function analysis; GMV, gray matter volume; HC, healthy control; KEGG, kyoto encyclopedia of genes and genomes; MC, mean curvature; MS, multiple sclerosis; MIND, Morphometric INverse Divergence; NMOSD, neuromyelitis optica spectrum disorders; PLS, partial least squares; SA, surface area; SD, sulcal depth.

## 2 Methods

### 2.1 Participant and MRI Data Preprocessing

We recruited MS patients based on the 2017 revised McDonald criteria [13] and NMOSD patients according to the 2015 NMOSD revised diagnostic criteria [2], as well as healthy controls. Participants were screened to exclude comorbid neurological or psychiatric disorders, and no overlap between MS and NMOSD diagnoses was present in the patient cohort. Imaging data were acquired on a 3.0 T MRI using a standardized protocol. All images underwent rigorous visual quality assessment, and only scans free of motion or other artifacts were included for further analysis. The full methodological details for lesion segmentation and filling, along with the structural preprocessing pipeline, have been described in our previous work [14]. Briefly, white matter lesions were automatically segmented using LST‐LPA [15] and subsequently filled on 3D T1‐weighted images using the SLF toolbox [16] to reduce lesion‐related bias.

### 2.2 Clinical Neuropsychological Assessments

Clinical variables included disease duration and neurological disability as assessed by the Expanded Disability Status Scale (EDSS) [17]. Verbal learning was assessed using the California Verbal Learning Test (CVLT‐II) [18], with the Total Learning score (sum of Trials 1–5) used as the representative measure. Visuospatial memory was evaluated with the Brief Visuospatial Memory Test‐Revised (BVMT‐Revised) [19], with the total score (sum of Trials 1–3) used as the representative measure. Verbal fluency was assessed with the Controlled Oral Word Association Test (COWAT), supplemented by a semantic fluency task [20]. Attention and information processing speed were measured using the Symbol Digit Modalities Test (SDMT) [21]. Visuospatial perception was assessed using the Judgment of Line Orientation (JLO) [22]. Depressive symptoms were assessed using the Beck Depression Inventory (BDI) [23].

### 2.3 Construction of MIND Network

To reduce parcel‐size variability, the DK‐308 atlas was generated from the conventional DK‐68 atlas using a backtracking algorithm, resulting in 308 spatially contiguous cortical parcels. Individual‐level parcellations were obtained by mapping the DK‐308 atlas onto each subject's cortical surface. For each region, five morphometric features—cortical thickness, volume, surface area, mean curvature, and sulcal depth—were extracted and normalized across the cortex to ensure comparable scales. MIND matrices (308 × 308) were then computed by calculating the Kullback–Leibler divergence between all pairs of regions, representing inter‐regional morphometric similarity. Regional MIND values were obtained by averaging all pairwise similarities across the cortex, without applying any thresholding.

### 2.4 Construction of MIND Gradients

The principal gradient derived from MIND networks represents a biologically meaningful axis of cortical organization, rather than a generic statistical variance component, as it captures a continuous progression of morphometric similarity from unimodal sensory/motor regions to transmodal association areas. Gradient analyses derived from MIND‐based structural similarity matrices were then generated with the BrainSpace toolbox (http://github.com/MICA‐MNI/BrainSpace) [7]. Firstly, consistent with previous study [7], the input matrix was made sparse (to 10% sparsity). To assess the robustness of the resulting gradient solution to the choice of sparsity threshold, we additionally repeated the analysis using alternative thresholds of 5% and 15% sparsity. Second, a positive 308 × 308 symmetric affinity matrix was computed using the normalized angle kernel to capture the morphological profile similarities of each region. Specifically, the cosine similarity between the morphological profiles of regions *i* and *j* was first calculated as:

$$S\left(i,j\right)=\text{cossim}\left(x_{i},x_{j}\right)=\frac{x_{i}x_{j}^{T}}{\left\|x_{i}\|\|x_{j}\right\|}$$

The resulting similarity matrix was subsequently transformed into an affinity matrix using the normalized angle kernel:

$$A\left(i,j\right)=1-\frac{\cos^{-1}\left[\text{cossim}\left(x_{i},x_{j}\right)\right]}{\pi }$$

Third, a symmetric affinity matrix was then fed into the diffusion map embedding (DME) algorithm, which is relatively insensitive to noise and computationally inexpensive. Notably, the hyperparameter α in the DME algorithm controls the influence of the density of sampling points on the manifold. Consistent with prior researches [24, 25, 26, 27], we set α = 0.5, as this value helps preserve the global relationships among data points in the embedding space and is relatively robust and less sensitive to noise in the covariance matrix. Fourth, an overall MIND gradient generated from all patients and controls, as a template, was applied to align individual gradients with Procrustes rotation. The gradient component explaining the greatest proportion of variance was selected for subsequent analyses. The group‐averaged primary gradient maps were created to represent group‐level gradient scores and to characterize the cortical morphometric hierarchy organization in each group.

### 2.5 Case–Control Analysis of the Regional MIND Gradient

Regional MIND gradient values were first residualized with respect to age, age^2^, sex, and total intracranial volume (TIV). The residualized values were then compared between patients with MS or NMOSD and HCs using two‐sided *t*‐tests. The comparison was performed separately for each ROI using a univariate ROI‐wise approach. Nominal *p‐*values were reported, with FDR correction applied for multiple‐comparison adjustment.

### 2.6 Correlations Between MIND Gradients and Clinical Variables

Spearman correlation analyses were performed to examine the associations between MIND gradient scores and clinical variables separately in the MS and NMOSD groups. Clinical measures were adjusted for age, age^2^, and sex, while gradient scores were additionally adjusted for TIV. Multiple‐comparison correction was then performed at two levels. First, for each selected clinical measure, FDR correction was applied across the 308 cortical ROIs. Second, as a more conservative sensitivity analysis, FDR correction was applied across the complete set of ROI‐by‐clinical‐measure tests involving all domain‐selected measures. Associations surviving only the within‐measure FDR correction were considered exploratory if they did not remain significant under the global FDR correction.

### 2.7 Spatial Correlations Between MIND Gradient and Neurotransmitter Maps

We examined spatial associations between the group‐averaged primary gradient maps and cortical distributions of 19 neurotransmitter receptors (https://github.com/netneurolab/hansen_receptors) via Spearman correlation analysis [28]. Statistical significance was determined using 10,000 spin permutation tests, and multiple comparisons across the 19 neurotransmitter systems were corrected using *q*_*FDR*_ < 0.05.

### 2.8 Preprocessing of Brain Transcriptomic Data

Brain transcriptional data were obtained from the Allen Human Brain Atlas (AHBA, http://human.brain‐map.org), comprising normalized microarray‐based gene expression profiles across 3702 spatially annotated tissue samples obtained from six postmortem human brains [29]. The AHBA dataset was processed using the abagen‐based Python toolbox (https://github.com/rmarkello/abagen) [30, 31]. The preprocessing pipeline followed a standardized workflow, including: (i) updating probe‐to‐gene annotations; (ii) filtering out probes with expression intensities below background noise in more than 50% of the samples; (iii) retaining the representative probe with the highest regional specificity for genes; (iv) matching tissue samples to brain regions; and (v) normalizing gene expression values using a scaled robust sigmoid (SRS) function. Due to the limited number of right‐hemisphere samples (*n* = 2) in the AHBA, analyses were confined to the left hemisphere, yielding a region‐by‐gene expression matrix of 15,632 genes across 152 cortical regions.

### 2.9 Transcription‐Neuroimaging Association Analysis

To investigate the transcriptional correlates of group‐level MIND gradient in MS and NMOSD, we applied partial least squares (PLS) regression. The predictor variables consisted of normalized gene expression profiles (15,632 genes × 152 cortical regions), while the response variables were group‐level gradients in MS and NMOSD across the same 152 regions. To assess whether the variance explained by the PLS component exceeded chance levels, we conducted a spatial permutation test (2000 iterations) using the Brain Surrogate Maps with Autocorrelated Spatial Heterogeneity package (BrainSMASH, https://github.com/murraylab/brainsmash) to generate spatial autocorrelation‐preserving surrogate maps [32].

The stability and significance of gene weights were further evaluated via bootstrapping with 5000 resamples, from which Z values were calculated by dividing each gene's weight by its bootstrap‐estimated standard error. The PLS component that maximized gene expression‐phenotype covariance explained > 10% of the variance and reached significance (*P*_perm_ < 0.05) was retained. Among these components, the one explaining the largest proportion of variance was selected for subsequent analyses. Genes surviving FDR correction (*q*_FDR_ < 0.05) were used for downstream analyses.

### 2.10 Functional Annotations of Associated Genes

To further explore the biological functions of these significant PLS2+ and PLS2‐ genes, we examined gene ontology biological processes (GO‐BP), gene ontology cellular components (GO‐CC), gene ontology molecular functions (GO‐MF) and Kyoto Encyclopedia of Genes and Genomes (KEGG) pathway analyses, with statistical significance set at *q*_FDR_ < 0.05. Results were analyzed and visualized via an online bioinformatics platform (https://www.bioinformatics.com.cn). To further investigate the cellular enrichment and developmental enrichment associated with MIND gradient in cortex, we applied the Specific Expression Analysis (SEA, http://doughertytools.wustl.edu/CSEAtool.html) tool [33], using the FDR threshold *q*_FDR_ < 0.05 and a pSI threshold of 0.05.

## 3 Results

### 3.1 Demographic Characteristics of Participants

A total of 62 MS patients (mean age 37.44 ± 11.58 years; 25 males and 37 females), 53 NMOSD patients (mean age 47.57 ± 14.24 years; 7 males and 46 females), and 74 healthy controls (mean age 48.23 ± 9.79 years; 19 males and 55 females) were included in this study.

### 3.2 Case–Control Differences in the Primary MIND Gradient

The first gradient (primary gradient) accounted for the largest proportion of variance in the MIND network (18.01%) and was therefore used for subsequent analyses (Supplementary Figure 1). The group‐averaged primary gradient maps generally followed a similar spatial organization, spanning primary to transmodal regions across the MS and NMOSD groups (Figure 2a,b and Supplementary Table 1). The first gradient solutions derived using 5% and 15% sparsity showed high correspondence with those obtained using the primary 10% sparsity threshold across all participants (Supplementary Figure 2), supporting the robustness of the MIND gradient to variations in sparsity threshold.

**FIGURE 2:**
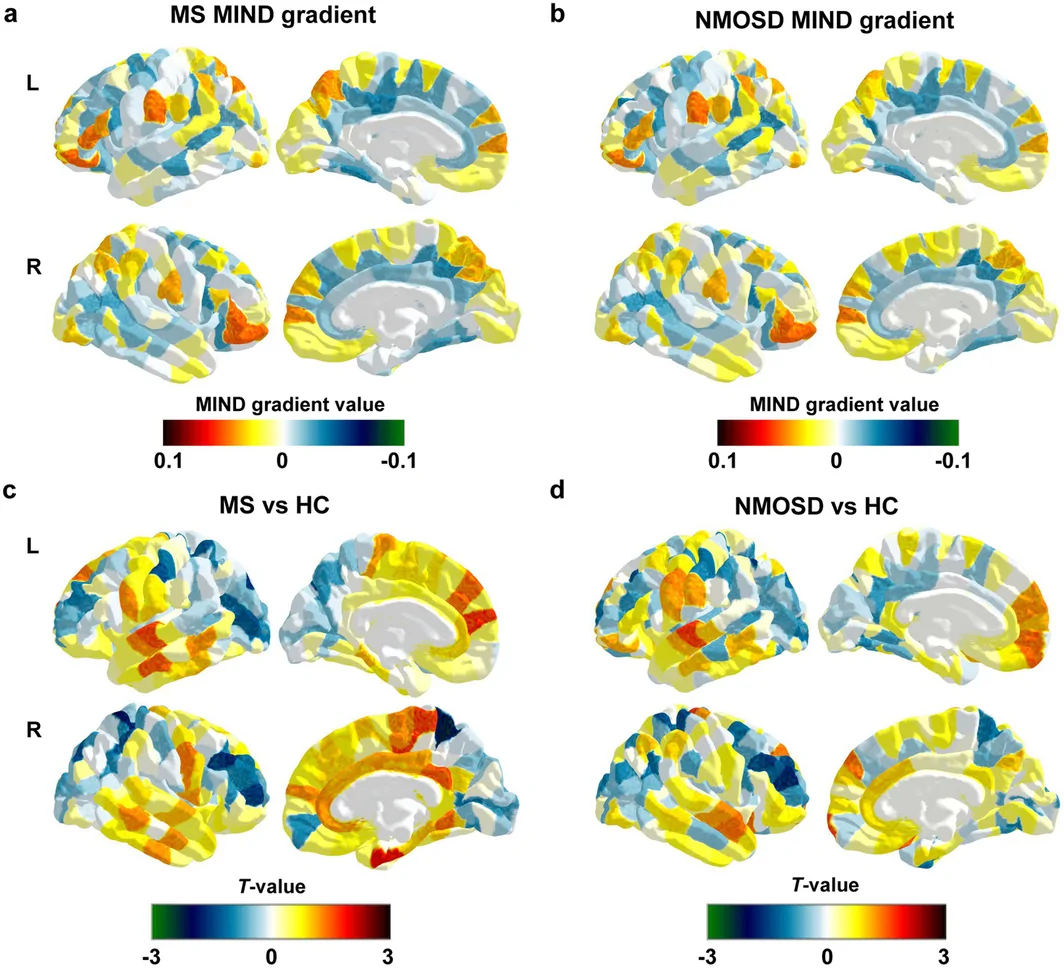
Primary MIND gradient and case–control differences in MS and NMOSD. (a–b) The distribution of primary MIND gradient in patients with MS and NMOSD. The color bar represents the primary MIND gradient value. (c–d) Cortical t‐maps illustrate the group differences between MS and HC and between NMOSD and HC. HC, healthy control; MIND, Morphometric INverse Divergence; MS, multiple sclerosis; NMOSD, neuromyelitis optica spectrum disorders.

The case–control differences suggested alterations along the primary MIND gradient in MS and NMOSD (Figure 2c,d, Supplementary Tables 2 and 3). Compared with HC, MS patients showed nominally significant alterations in six ROIs (*p* < 0.05), including three ROIs with decreased and three ROIs with increased gradient values. Decreased gradient values were observed in the left inferior parietal cortex (part 4; *T* = −1.999, *p* = 0.048), right precuneus (part 3; *T* = −2.689, *p* = 0.008), and right superior parietal cortex (part 6; *T* = −2.331, *p* = 0.021), whereas increased gradient values were observed in the left superior frontal cortex (part 3; *T* = 1.983, *p* = 0.049), right entorhinal cortex (part 1; *T* = 2.215, *p* = 0.029), and right paracentral cortex (part 3; *T* = 1.993, *p* = 0.048). Similarly, compared with HC, NMOSD patients showed nominally significant alterations in two ROIs, both of which exhibited decreased gradient values, including the left supramarginal cortex (part 1; *T* = −2.034, *p* = 0.044) and right rostral middle frontal cortex (part 9; *T* = −2.010, *p* = 0.047). None of these findings survived FDR correction.

In addition, we characterized the regional alteration profiles based on the direction of case–control effects, without applying statistical significance thresholds. We identified 106 regions showing concordant increases, 95 regions showing concordant decreases, and 107 regions exhibiting opposite directions of alteration between MS and NMOSD (Supplementary Figure 3). We further examined the overlap of statistically supported alterations by identifying cortical regions that showed nominal significance (*p* < 0.05) in both the MS versus HC and NMOSD versus HC comparisons. Although no regions met this overlap criterion, this finding may partly reflect the relatively subtle effect sizes of case–control differences, as well as the limited ability of the current sample size to detect smaller overlapping effects.

### 3.3 Regional Differences in the Primary MIND Gradient Between MS and NMOSD

We performed ROI‐wise comparisons directly contrasting MS and NMOSD using the same covariates as in the original analyses, with FDR correction applied across all cortical regions. No cortical regions survived FDR correction in the direct MS‐NMOSD comparison. At the nominal significance level (*p* < 0.05), three regions showed potential between‐disease differences, including the left precentral part 5 (*T* = −2.056, *p* = 0.042), right inferior parietal part 4 (*T* = −2.306, *p* = 0.023), and right supramarginal part 3 (*T* = −2.188, *p* = 0.031) (Supplementary Figure 4 and Supplementary Table 4).

### 3.4 Association of Clinical Features With the Primary MIND Gradient

The relationships across the primary MIND gradient and clinical outcomes showed distinct patterns in MS groups and NMOSD groups. Under region‐wise FDR correction applied separately within each clinical measure, higher gradient scores in the left middle temporal region (part3: rho = 0.598, *q*_*FDR*_ = 0.032), left superior frontal region (part13: rho = 0.574, *q*_*FDR*_ = 0.045), right precentral region (part5: rho = 0.607, *q*_*FDR*_ = 0.032), and right superior frontal region (part13: rho = 0.597, *q*_*FDR*_ = 0.032) were positively associated with COWAT scores in the MS group. In the NMOSD group, gradient scores in the left postcentral region (part1: rho = −0.551, *q*_*FDR*_ = 0.028) and right fusiform region (part3: rho = 0.541, *q*_*FDR*_ = 0.028) were associated with semantic fluency scores. However, none of these associations remained significant after applying the more stringent FDR correction across the full set of ROI‐by‐clinical‐measure tests.

### 3.5 Transcriptomic Correlates of the Primary MIND Gradient

Based on the transcriptional matrix of brain tissues, we performed PLS regression to identify gene expression patterns underlying the neuroanatomical distribution of the primary MIND gradient in MS and NMOSD. In both groups, the second component (PLS2) significantly explained the largest proportion of variance (21.94% in MS; 24.67% in NMOSD, *P*_perm_ < 0.05) (Figure 3a,b). The spatial distribution of PLS2‐weighted gene expression maps showed significant correlations with the primary MIND gradient in both groups, with evidence of spatial correspondence between cortical gradient alterations and transcriptomic patterns in MS (*r* = 0.468, *P*_perm_ = 0.036) and NMOSD (*r* = 0.497, *P*_perm_ = 0.011) (Figure 3c,d). Specifically, *P2RY12* exhibited the highest Z‐scores in both MS and NMOSD (7.07 and 7.50, respectively), whereas *ABTB2* showed the lowest Z‐scores (−6.71 and −7.17, respectively) (Figure 3e,f).

**FIGURE 3:**
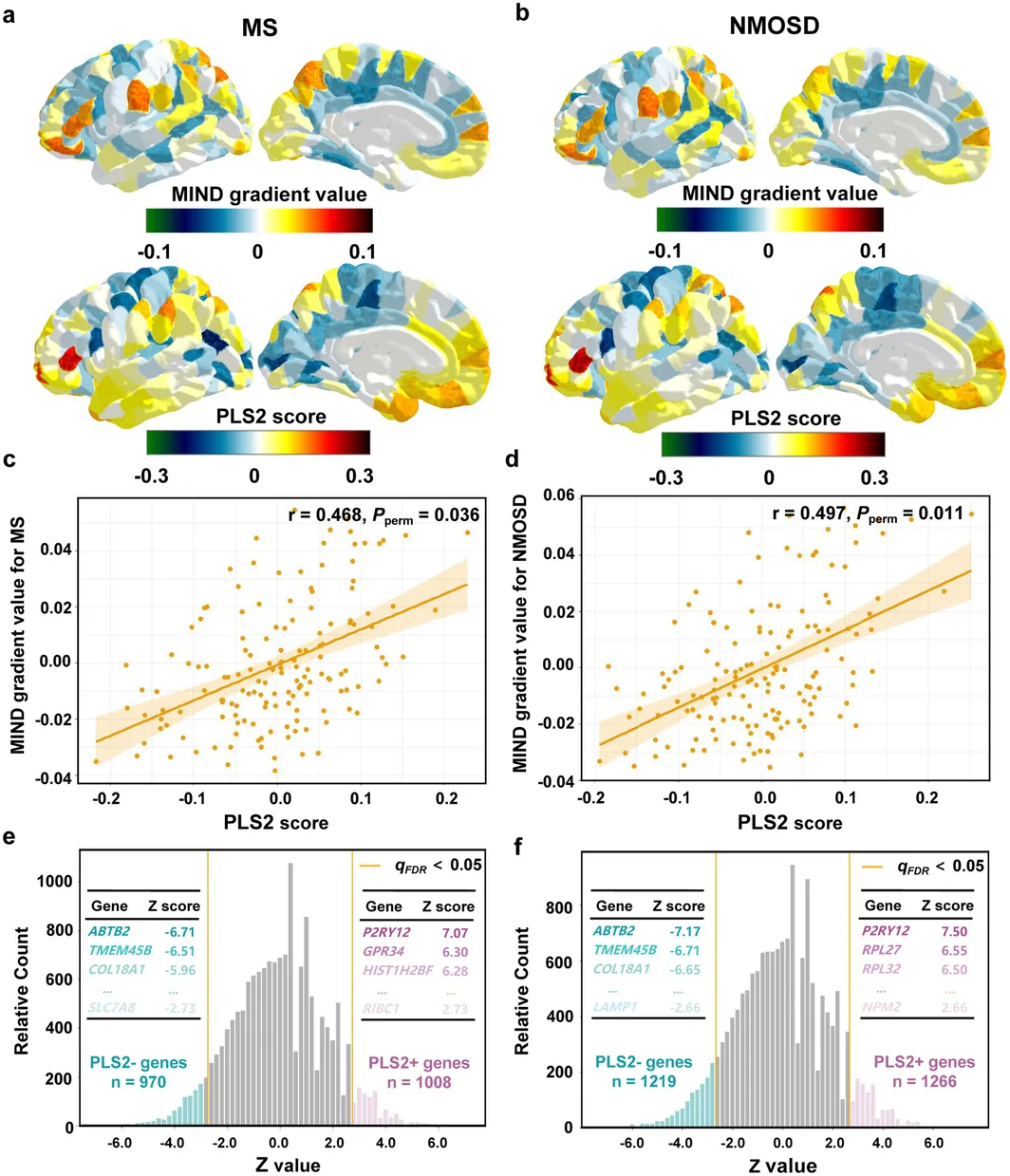
Gene expression patterns related to the primary MIND gradient. (a–b) The distribution of primary gradient and PLS2 scores in the left hemisphere of MS group (a) and NMOSD group (b). The upper color bar represents the primary MIND gradient value, while the lower color bar represents the PLS2 score. (c–d) The density scatter plots show the associations between regional PLS2 scores and MIND gradient for the MS group (c) and NMOSD group (d). The shaded area indicates the 95% confidence interval. (e–f) Significant PLS2‐weighted genes identified in MS (e) and NMOSD (f). The table displays ranked PLS2 genes based on Z score. FDR, false discovery rate; MIND, Morphometric INverse Divergence; MS, multiple sclerosis; NMOSD, neuromyelitis optica spectrum disorders; PLS, partial least squares; perm, permutation.

To further assess the robustness of the PLS models, we performed an additional spatial cross‐validation analysis. For each diagnostic group (MS and NMOSD), 25% of cortical regions were randomly excluded from the original 152 regions, and the PLS model was re‐fitted using the remaining 114 regions. The resulting PLS‐derived regional loading patterns from each of the 20 iterations were then used to reconstruct regional PLS scores across the full set of cortical regions, and their reproducibility was evaluated using Spearman correlations. The PLS‐derived spatial patterns demonstrated high stability across iterations. In the MS group, the correlation coefficients across the 20 iterations showed a mean Spearman correlation of rho = 0.868 ± 0.040. Similarly, in the NMOSD group, the PLS patterns remained highly reproducible, with a mean Spearman correlation of rho = 0.861 ± 0.042 (Supplementary Figure 5). These findings indicate that the observed transcriptomic‐gradient associations were robust to regional resampling.

### 3.6 Functional Annotation of MS‐ and NMOSD‐Associated Genes

We identified 1008 PLS2+ genes and 970 PLS2‐ genes for MS, along with 1266 PLS2+ genes and 1219 PLS2‐ genes for NMOSD (all *q*_FDR_ < 0.05, Supplementary Tables 5 and 6). These genes showed covariation with regional values of the primary MIND gradient in MS and NMOSD. To further elucidate the molecular pathways underlying genes associated with regional variations in the MIND primary gradient, we performed GO‐BP, GO‐CC, GO‐MF, and KEGG pathway enrichment analyses (Figures 4 and 5; Supplementary Table 7).

**FIGURE 4:**
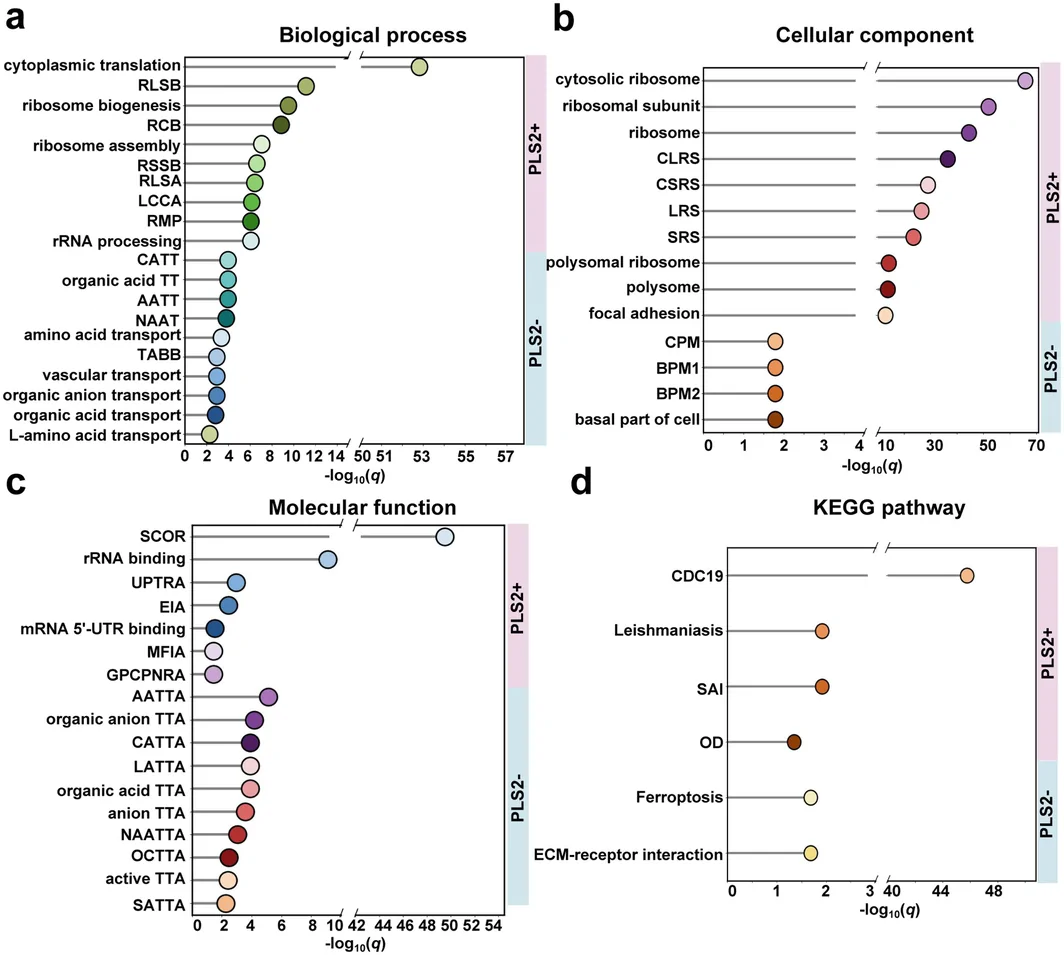
Functional enrichment analysis of the PLS2 genes in the MS group. (a–c) Lollipop plots illustrating Gene Ontology (GO) enrichment results for up to the top 10 significantly enriched terms in each GO category based on PLS2‐positive and PLS2‐negative genes in the MS group. (d) The KEGG pathways of PLS2 genes in the MS group. Statistical significance is shown on the x‐axis as −log_10_ (q) (q_FDR_ < 0.05). AATT, amino acid transmembrane transport; AATTA, amino acid transmembrane transporter activity; anion TTA, anion transmembrane transporter activity; active TTA, active transmembrane transporter activity; BPM1, basal plasma membrane; BPM2, basolateral plasma membrane; CATT, carboxylic acid transmembrane transport; CATTA, carboxylic acid transmembrane transporter activity; CDC19, Coronavirus disease COVID‐19; CLRS, cytosolic large ribosomal subunit; CPM, cell projection membrane; CSRS, cytosolic small ribosomal subunit; EIA, enzyme inhibitor activity; GPCPNRA, G protein–coupled purinergic nucleotide receptor activity; KEGG, Kyoto Encyclopedia of Genes and Genomes; LATTA, L‐amino acid transmembrane transporter activity; LCCA, leukocyte cell–cell adhesion; LRS, large ribosomal subunit; MFIA, molecular function inhibitor activity; MS, multiple sclerosis; NAAT, neutral amino acid transport; NAATTA, neutral amino acid transmembrane transporter activity; organic acid TT, organic acid transmembrane transport; organic anion TTA, organic anion transmembrane transporter activity; organic acid TTA, organic acid transmembrane transporter activity; OCTTA, organic cation transmembrane transporter activity; OD, osteoclast differentiation; PLS, partial least squares; RCB, ribonucleoprotein complex biogenesis; RLSA, ribosomal large subunit assembly; RLSB, ribosomal large subunit biogenesis; RMP, rRNA metabolic process; RSSB, ribosomal small subunit biogenesis; SAI, Staphylococcus aureus infection; SATTA, secondary active transmembrane transporter activity; SCOR, structural constituent of ribosome; SRS, small ribosomal subunit; TABB, transport across blood–brain barrier; UPTRA, ubiquitin–protein transferase regulator activity.

**FIGURE 5:**
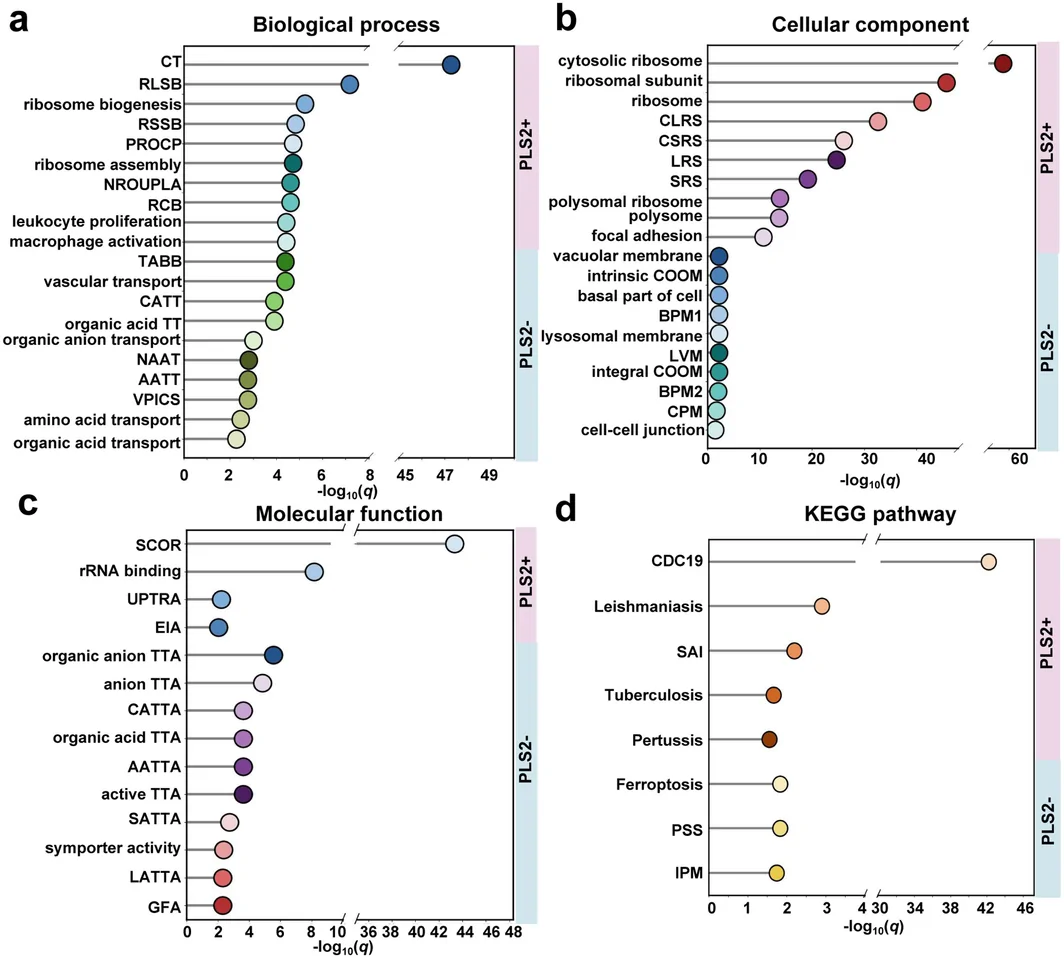
Functional enrichment analysis of the PLS2 genes in the NMOSD group. (a–c) Lollipop plots illustrating Gene Ontology (GO) enrichment results for up to the top 10 significantly enriched terms in each GO category based on PLS2‐positive and PLS2‐negative genes in the NMOSD group. (d) The KEGG pathways of PLS2 genes in the NMOSD group. Statistical significance is shown on the x‐axis as −log_10_ (q) (q_FDR_ < 0.05). AATT, amino acid transmembrane transport; AATTA, amino acid transmembrane transporter activity; anion TTA, anion transmembrane transporter activity; active TTA, active transmembrane transporter activity; BPM1, basal plasma membrane; BPM2, basolateral plasma membrane; CATT, carboxylic acid transmembrane transport; CATTA, carboxylic acid transmembrane transporter activity; CDC19, Coronavirus disease COVID‐19; CLRS, cytosolic large ribosomal subunit; CSRS, cytosolic small ribosomal subunit; CT, cytoplasmic translation; CPM, cell projection membrane; EIA, enzyme inhibitor activity; GFA, glycerophospholipid flippase activity; intrinsic COOM, intrinsic component of organelle membrane; integral COOM, integral component of organelle membrane; IPM, inositol phosphate metabolism; KEGG, Kyoto Encyclopedia of Genes and Genomes; LATTA, L‐amino acid transmembrane transporter activity; LVM, lytic vacuole membrane; LRS, large ribosomal subunit; NAAT, neutral amino acid transport; NMOSD, neuromyelitis optica spectrum disorder; NROUPLA, negative regulation of ubiquitin protein ligase activity; organic acid TT, organic acid transmembrane transport; organic anion TTA, organic anion transmembrane transporter activity; organic acid TTA, organic acid transmembrane transporter activity; PLS, partial least squares; PROCP, positive regulation of cytokine production; PSS, phosphatidylinositol signaling system; RCB, ribonucleoprotein complex biogenesis; RLSB, ribosomal large subunit biogenesis; RSSB, ribosomal small subunit biogenesis; SAI, Staphylococcus aureus infection; SATTA, secondary active transmembrane transporter activity; SCOR, structural constituent of ribosome; SRS, small ribosomal subunit; TABB, transport across blood–brain barrier; UPTRA, ubiquitin‐protein transferase regulator activity; VPICS, vascular process in circulatory system.

#### 3.6.1 GO Enrichment Analysis for PLS2‐Associated Genes in MS and NMOSD Groups

For MS PLS2 genes, PLS2+ genes exhibited significant enrichment in biological processes of immune and inflammation‐related pathways, including immune activation (regulation of T cell activation: *q*_*FDR*_ = 1.912 × 10^−5^; myeloid leukocyte activation: *q*_*FDR*_ = 1.736 × 10^−5^, interleukin‐6 production: *q*_*FDR*_ = 4.471 × 10^−4^), and antigen processing and presentation of peptide antigen via MHC Class II (*q*_*FDR*_ = 0.024), along with regulation of phagocytosis (*q*_*FDR*_ = 1.462 × 10^−4^) and response to axon injury (*q*_*FDR*_ = 0.036). For the cellular component, PLS2+ genes were enriched in synaptic structures such as neuron to neuron synapse (*q*_*FDR*_ = 1.126 × 10^−5^) and asymmetric synapse (*q*_*FDR*_ = 3.189 × 10^−5^). MS PLS2‐ genes were enriched in several biological pathways, including transport across the blood–brain barrier (*q*_*FDR*_ = 0.001), amino acid transport (*q*_*FDR*_ = 4.470 × 10^−4^), and carboxylic acid transport (*q*_*FDR*_ = 0.008), and vascular transport (*q*_*FDR*_ = 0.001).

For NMOSD PLS2 genes, PLS2+ genes in NMOSD highlight a combined signature of B‐cell–linked immune activation, myeloid‐mediated inflammation, and synaptic and axonal processes, including positive regulation of cytokine production (*q*_*FDR*_ = 1.884 × 10^−5^), regulation of B cell proliferation (*q*_*FDR*_ = 0.012), myeloid leukocyte activation (*q*_*FDR*_ = 7.964 × 10^−5^), and macrophage activation (*q*_*FDR*_ = 3.671 × 10^−5^). In addition, PLS2+ genes were also enriched in synaptic structures, including neuron to neuron synapse (*q*_*FDR*_ = 2.700 × 10^−7^), asymmetric synapse (*q*_*FDR*_ = 1.446 × 10^−6^), and glutamatergic synapse (*q*_*FDR*_ = 0.009). For NMOSD PLS2‐ genes, biological process enrichment was dominated by pathways related to substance transport and transmembrane movement, including transport across blood–brain barrier (*q*_*FDR*_ = 4.077 × 10^−5^), vascular transport (*q*_*FDR*_ = 4.077 × 10^−5^) and amino acid transmembrane transport (*q*_*FDR*_ = 0.002). Consistently, cellular component enrichment pointed primarily to membrane‐associated structures, including basal plasma membrane (*q*_*FDR*_ = 0.006) and integral component of organelle membrane (*q*_*FDR*_ = 0.007), while molecular function highlighted transporter, symporter, and antiporter activities, including organic anion transmembrane transporter activity (*q*_*FDR*_ = 2.753 × 10^−6^) and symporter activity (*q*_*FDR*_ = 0.004).

#### 3.6.2 KEGG Pathway Analysis for PLS2‐Associated Genes in MS and NMOSD Groups

The MS PLS2+ genes were enriched in Coronavirus disease‐COVID‐19 pathway (*q*_*FDR*_ = 1.692 × 10^−46^) and osteoclast differentiation (*q*_*FDR*_ = 0.045), which contain genes associated with immune activation and cytokine storm responses, indirectly mapping onto the inflammatory modules observed in MS. The MS PLS2‐ genes were significantly enriched in ferroptosis and ECM‐receptor interaction pathways (both *q*_*FDR*_ = 0.021).

In NMOSD, PLS2+ genes were significantly enriched in pathways related to infectious and immune diseases, including coronavirus disease‐COVID‐19 (*q*_*FDR*_ = 6.354 × 10^−43^), *Staphylococcus aureus* infection (*q*_*FDR*_ = 0.006), tuberculosis (*q*_*FDR*_ = 0.022), pertussis (*q*_*FDR*_ = 0.028), and leishmaniasis (*q*_*FDR*_ = 0.001). For NMOSD PLS2‐ genes, ferroptosis (*q*_*FDR*_ = 0.015) and phosphatidylinositol signaling system (*q*_*FDR*_ = 0.015) are associated with NMOSD.

### 3.7 Shared and Divergent Biological Processes

We performed a comparative analysis of the GO‐BP enrichment profiles of the MS‐ and NMOSD‐associated gene sets and identified both shared and divergent enrichment patterns. A total of 123 GO‐BP terms were significantly enriched in both disorders, whereas 73 terms were significant only in MS and 24 only in NMOSD. Terms significant only in MS included selected processes related to immune‐cell activation, leukocyte migration and adhesion, MAPK/ERK signaling, and p53‐related signaling. Terms significant only in NMOSD included several lipid metabolic processes, such as glycerolipid, glycerophospholipid, membrane lipid, glycolipid, and sphingolipid metabolism.

### 3.8 Cell‐Type Enrichment Analysis of Associated Genes

Cell‐type enrichment analysis demonstrated that MS and NMOSD PLS2‐ genes showed significant enrichment predominantly in myelinating oligodendrocytes of the cortex (MS, *q*_*FDR*_ = 1.605 × 10^−4^; NMOSD, *q*_*FDR*_ = 1.027 × 10^−4^, Figure 6a and Supplementary Table 8).

**FIGURE 6:**
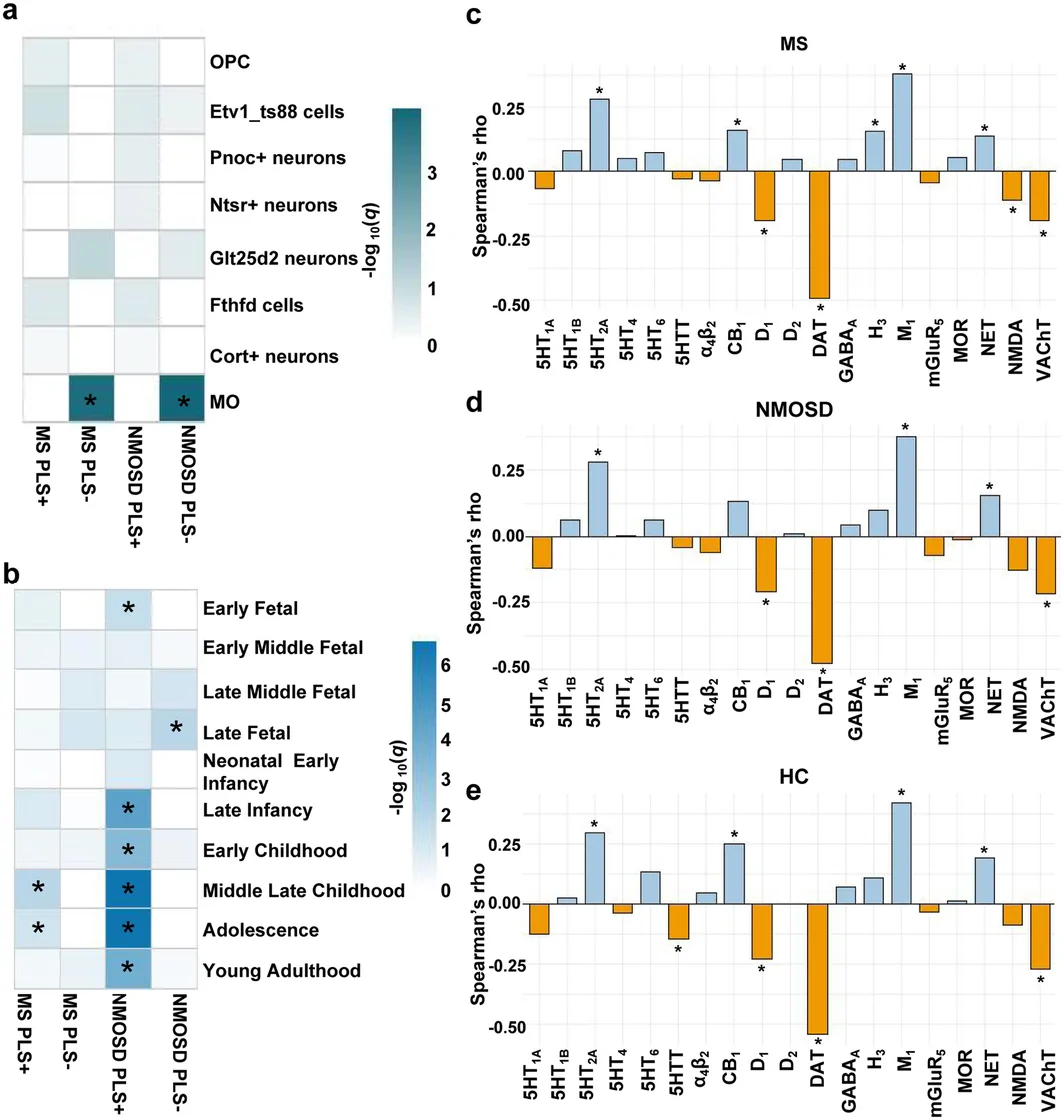
Cell‐type specificity, developmental trajectories, and neurotransmitter associations. (a) Specific expression analysis across cell types for MS and NMOSD. The color bar shows the −log_10_ (q) value. (b) Specific temporal windows for MS and NMOSD in the cortex. The color bar shows the −log_10_ (q) value. (c–e). Spatial association between primary MIND gradient and receptors/transporters densities in MS, NMOSD and HC. The Asterisks (*) denote significance at q_FDR_ < 0.05. α_4_β_2_, nicotinic acetylcholine receptor α_4_β_2_; CB1, cannabinoid receptor 1; D1, dopamine D1 receptor; D2, dopamine D2 receptor; DAT, dopamine transporter; GABA_A_, gamma‐aminobutyric acid receptor A; H_3_, histamine H_3_ receptor; HC, healthy control; M_1_, muscarinic acetylcholine receptor M_1_; mGluR5, metabotropic glutamate receptor 5; MOR, μ‐opioid receptor; MS, multiple sclerosis; MO, myelinating oligodendrocyte; NET, norepinephrine transporter; NMDA, N‐methyl‐D‐aspartate receptor; NMOSD, neuromyelitis optica spectrum disorders; OPC, oligodendrocyte progenitor cells; PLS, partial least squares; 5‐HT_1A_, 5‐hydroxytryptamine receptor 1A; 5‐HT_1B_, 5‐hydroxytryptamine receptor 1B; 5‐HT_2A_, 5‐hydroxytryptamine receptor 2A; 5‐HT_4_, 5‐hydroxytryptamine receptor 4; 5‐HT_6_, 5‐hydroxytryptamine receptor 6; 5‐HTT, 5‐hydroxytryptamine transporter; VAChT, vesicular acetylcholine transporter.

### 3.9 Temporal Enrichment Analysis of Associated Genes

We evaluated the spatiotemporal gene expression patterns for MS and NMOSD through brain regional and developmental SEA analysis, spanning from early fetal stages to young adulthood (Figure 6b and Supplementary Table 9). The cortical regions of enriched time windows in MS PLS2+ genes exerted effects in middle late childhood (*q*_*FDR*_ = 0.011) and adolescence (*q*_*FDR*_ = 0.049), coinciding with cortical myelination and synaptic remodeling. In contrast, NMOSD PLS2+ genes showed significant enrichment during the early fetal (*q*_*FDR*_ = 0.024), late infancy (*q*_*FDR*_ = 3.327 × 10^−5^), middle late childhood (*q*_*FDR*_ = 2.346 × 10^−7^), early childhood (*q*_*FDR*_ = 4.559 × 10^−4^), adolescence (*q*_*FDR*_ = 2.346 × 10^−7^), and young adulthood stages (*q*_*FDR*_ = 1.393 × 10^−4^). NMOSD PLS2‐ genes showed significant enrichment in late fetal (*q*_*FDR*_ = 0.011).

### 3.10 Associations Between Neurotransmitter Receptor/Transporter Distributions and the MIND Gradient

The primary MIND gradient in MS was correlated with the spatial distribution of 5HT_2A_ (rho = 0.282, *q*_*FDR*_ < 0.05), CB_1_ (rho = 0.161, *q*_*FDR*_ < 0.05), D_1_ (rho = −0.190, *q*_*FDR*_ < 0.05), DAT (rho = −0.493, *q*_*FDR*_ < 0.05), H_3_ (rho = 0.157, *q*_*FDR*_ < 0.05), M1 (rho = 0.379, *q*_*FDR*_ < 0.05), NET (rho = 0.139, *q*_*FDR*_ < 0.05), NMDA (rho = −0.111, *q*_*FDR*_ < 0.05), and VAChT (rho = −0.189, *q*_*FDR*_ < 0.05) (Figure 6c).

In the NMOSD group, the primary MIND gradient demonstrated a significant spatial correspondence with the distribution of 5HT_2A_ (rho = 0.279, *q*_*FDR*_ < 0.05), D_1_ (rho = −0.207, *q*_*FDR*_ < 0.05), DAT (rho = −0.478, *q*_*FDR*_ < 0.05), M_1_ (rho = 0.375, *q*_*FDR*_ < 0.05), NET (rho = 0.155, *q*_*FDR*_ < 0.05), and VAChT (rho = −0.216, *q*_*FDR*_ < 0.05) (Figure 6d).

In the HC group, the primary MIND gradient was significantly correlated with the spatial distributions of 5HT_2A_ (rho = 0.295, *q*_*FDR*_ < 0.05), 5HTT (rho = −0.144, *q*_*FDR*_ < 0.05), CB_1_ (rho = 0.249, *q*_*FDR*_ < 0.05), D_1_ (rho = −0.230, *q*_*FDR*_ < 0.05), DAT (rho = −0.541, *q*_*FDR*_ < 0.05), M_1_ (rho = 0.421, *q*_*FDR*_ < 0.05), NET (rho = 0.194, *q*_*FDR*_ < 0.05), and VAChT (rho = −0.270, *q*_*FDR*_ < 0.05) (Figure 6e).

## 4 Discussion

In this study, we investigated cortical hierarchical disorganization and its transcriptional underpinnings in MS and NMOSD using a morphometry‐informed network‐based gradient framework. This integrative approach revealed subtle reconfigurations of cortical structural hierarchy in both disorders, including coexisting bidirectional gradient alterations that would not be captured by conventional methods. These gradients were further examined in relation to clinical measures, neurotransmitter distributions, spatial gene expression patterns, biological pathways, cell‐type enrichment, and developmental‐stage enrichment. By integrating these multiple levels of evidence, our findings provide a framework linking cortical organization with its molecular, functional, and developmental correlates, offering insights into the neurobiological features of demyelinating disorders and highlighting potential directions for future biomarker and therapeutic research.

By integrating the MIND network, which captures fine‐grained morphometric covariance at the vertex level, with gradient decomposition, we were able to characterize cortical hierarchy with greater anatomical specificity than conventional structural covariance or diffusion embedding approaches. At the regional level, alterations of the primary MIND gradient showed partially distinct spatial patterns across MS and NMOSD. In MS, altered gradient values were mainly observed in the precuneus and inferior/superior parietal regions, which are key hubs for multimodal integration and network organization. These regions, which showed altered positioning along the primary cortical hierarchy, have been implicated in higher‐order cognitive processing [34]. Additionally, NMOSD showed alterations in the supramarginal and rostral middle frontal cortices, which are involved in cognitive control and integrative processing. Consistent with the involvement of these regions, a previous study has reported reduced cortical thickness in the bilateral rostral middle frontal gyrus in NMOSD compared with healthy controls [35]. Moreover, the supramarginal gyrus comprises functionally specialized subregions involved in phonological processing, auditory memory, and higher‐order cognitive control [36]. Together, these findings provide preliminary evidence that alterations of cortical hierarchical organization may involve different regional vulnerabilities across MS and NMOSD.

Importantly, the potential behavioral relevance of these hierarchical alterations was further examined through their region‐specific associations with cognitive performance. In MS, positive associations were observed between COWAT scores and gradient values in the left middle temporal, left superior frontal, right precentral, and right superior frontal regions. These findings suggest that individual differences in regional hierarchical organization may be related to verbal fluency in MS. In NMOSD, gradient values in the left postcentral and right fusiform gyrus were associated with semantic fluency scores. These regions are critically involved in language‐related processes [37, 38]. These findings suggest a potential relationship between regional hierarchical organization and verbal fluency performance in NMOSD. However, given the correlational nature of these findings, these associations should not be interpreted as evidence of causal relationships.

The significant spatial correspondence between the primary MIND gradient and cortical gene transcriptional profiles provides converging evidence that disruptions in morphometric cortical hierarchy are associated with underlying molecular architectures. PLS analysis of MS revealed *P2RY12* and *CX3CR1* as genes with strong positive weighting in MS. *P2RY12*, a microglia‐specific homeostatic marker, is characterized by its immunoreactivity in MS lesions and is markedly downregulated in activated or lesion‐associated microglia within MS brains [39, 40]. *CX3CR1*, a critical fractalkine receptor involved in neuron–microglia communication, plays a role in limiting toxic gene responses during neuroinflammation and has been implicated in cuprizone‐induced demyelination [41]. Moreover, CX3CR1^+^/UCHL1^+^ extracellular vesicles, serving as a potential blood biomarker, show strong potential for diagnosing MS and distinguishing it from other neurological disorders [42]. Besides, genetic studies have implicated *IL7R* in susceptibility or progression to MS [43, 44]. The PLS2‐significant genes in NMOSD included several key human leukocyte antigen (HLA) genes. The HLA regions, based on the importance of this region in the regulation of immune responses, have represented one of the important genetic susceptibility factors for NMOSD [45]. In a South Indian study, specific HLA DRB1‐DQB1 haplotypes were associated with susceptibility to NMO [46].

In both disorders, genes associated with the MIND gradient pattern were predominantly enriched in several immune‐inflammatory pathways, such as inflammatory response to antigenic stimulus, positive regulation of T cell proliferation and leukocyte proliferation. In MS, the enriched pathways included MAPK‐mediated T‐cell‐driven immune activation (ERK1 and ERK2 cascade, positive regulation of MAPK cascade). Dysregulated MAPK/ERK signaling in microglia has been implicated in MS pathophysiology [47]. MAPK signaling has also been implicated in cuprizone‐induced demyelination in mice [48]. In NMOSD, enrichment of GO terms related to MHC complexes and peptide antigen assembly suggested the involvement of antigen presentation. This finding may be relevant to AQP4‐specific T‐cell responses, which have been shown to influence CNS lesion localization in an experimental model of NMOSD [49].

On the other hand, the temporal enrichment of cortical regions associated with MS‐ and NMOSD‐related hierarchical disorganization revealed distinct developmental trajectories. The MS PLS2+ genes exhibited significant enrichment during middle late childhood and adolescence, overlapping with periods of ongoing white matter maturation and cortical myelination [50, 51]. In contrast, NMOSD PLS2+ genes showed enrichment at multiple developmental stages ranging from early fetal life to young adulthood, suggesting broader developmental involvement. Furthermore, the cellular enrichment analysis indicated that the PLS2‐ genes, enriched in myelinating oligodendrocytes, represented transcriptional programs supporting lipid metabolism, axonal ensheathment, and myelin maintenance, suggesting that their spatial pattern along the disease gradient may be related to alterations in oligodendrocyte and myelin integrity. Additionally, the spatial distributions of neurotransmitter receptors were associated with the cortical MIND gradient, suggesting a potential relationship between hierarchical morphometric organization and the spatial distribution of neurotransmitter systems across the studied groups.

Several limitations of this study should be illustrated. First, the relatively modest sample size may have limited the statistical power to detect regional between‐group differences. Accordingly, the observed regional patterns should be regarded as preliminary and interpreted with caution, particularly with respect to disease‐specific effects. Larger independent cohorts are needed to establish the robustness and reproducibility of these findings. In addition, although sex was included as a covariate in all relevant statistical models, residual confounding related to the unequal sex distribution across groups cannot be entirely excluded. Second, our transcriptomic analyses were restricted to 152 left‐hemisphere cortical regions because the AHBA provides relatively comprehensive cortical sampling predominantly in the left hemisphere. In addition, these analyses were based on postmortem transcriptomic data from six adult donors. Future studies integrating more comprehensive bilateral transcriptomic datasets and larger, more diverse donor samples are warranted to further validate these findings. Third, although associations between cortical gradients and cognitive performance were observed, these findings should be interpreted cautiously. The observed brain‐behavior associations may be influenced by limited statistical power, particularly given the modest sample sizes and the multiple‐comparison burden. In addition, cognitive performance is likely shaped by multiple factors, rather than cortical hierarchical organization alone. Accordingly, these findings should be considered exploratory and require validation in larger independent cohorts. Fourth, the neurotransmitter maps used in our analyses were derived from aggregated, group‐averaged normative PET datasets of healthy individuals and thus represent population‐level estimates rather than individual‐specific measures. While these normative maps provide a useful reference for characterizing the spatial distribution of neurotransmitter systems, they may not fully reflect interindividual variability or disease‐related changes. Fifth, a potential influence of cortical lesions on cortical surface reconstruction cannot be completely excluded. Future studies using high‐resolution MRI sequences optimized for cortical lesion detection are warranted. Finally, several clinically important measures, including EDSS and SDMT, did not show significant associations with cortical hierarchy alterations, likely because cortical hierarchical organization reflects distributed system‐level reconfiguration rather than focal lesion burden or overt disability, and such alterations may precede measurable clinical deterioration, limiting the sensitivity of cross‐sectional clinical assessments.

Future studies should further validate and extend these findings in larger, independent, and multi‐center cohorts with greater demographic and clinical diversity. Longitudinal studies will be particularly important for determining whether alterations in cortical hierarchical organization change with disease progression, treatment, or cognitive decline, and for clarifying the temporal relationships between cortical organization and clinical outcomes. Integrating longitudinal neuroimaging with genetic, transcriptomic, proteomic, and other molecular measures may further help clarify the biological correlates of cortical hierarchy alterations. In addition, incorporating treatment‐response data and clinical trial cohorts could help determine whether these neurobiological signatures have potential utility for patient stratification or treatment monitoring.

Across MS and NMOSD, MIND‐gradient analyses suggested regional alterations in cortical hierarchical organization and explored associations between regional gradient values and cognitive performance. Integrative transcriptomic analyses identified shared and divergent biological enrichment patterns, suggesting potential molecular correlates of regional MIND gradient values. Together, these findings highlight cortical hierarchical organization as a potential neurobiological feature of demyelinating diseases and provide a framework for investigating its relationships with clinical phenotypes and molecular processes.

## Author Contributions

**Yingying Xie and Shiqi Lin:** conceptualization, formal analysis, visualization, writing – original draft. **Jie Sun and Bohong Zou:** investigation, formal analysis. **Han Zhang, Jiaojiao Du and Zhang Zhang:** investigation. **Dairong Cao:** supervision, writing – review and editing. **Ningnannan Zhang:** supervision, investigation, writing – review and editing. All authors critically reviewed the manuscript.

## Funding

We acknowledge funding from the National Natural Science Foundation of China (82502309, 82302139, 82071907); Joint Funds for the Innovation of Science and Technology, Fujian Province (2025Y9214); China Postdoctoral Science Foundation (2025M781907); Chronic Disease Management Research Project of National Health Commission Capacity Building and Continuing Education Center (GWJJMB202510025082); Tianjin Health Research Project (TJWJ2023QN010); Scientific Research Planning Project of Tianjin Education Commission (2021KJ219); Wu Jieping Medical Foundation‐Special Fund for Clinical Research (320.6750.2022‐3‐5); Tianjin Key Medical Discipline Construction Project (TJYXZDXK‐3‐008C).

## Ethics Statement

This study was conducted in accordance with the ethical standards of the World Medical Association's Declaration of Helsinki and was approved by the Institutional Review Board of Tianjin Medical University General Hospital (approval number: IRB2020‐YX‐137‐01), and written informed consent was obtained from all participants.

## Conflicts of Interest

The authors declare no conflicts of interest.

## Supporting information


**Supplementary Figure 1.** Variance explained by the first ten gradient components.
**Supplementary Figure 2**. Correlations between gradient values obtained at 10% sparsity and those obtained at 5% and 15% sparsity. HC, healthy controls; MS, multiple sclerosis; NMOSD, neuromyelitis optica spectrum disorder.
**Supplementary Figure 3**. Cortical regions showing concordant and divergent patterns of MIND‐gradient alterations between MS and NMOSD. MS, multiple sclerosis; NMOSD, neuromyelitis optica spectrum disorder.
**Supplementary Figure 4**. Distribution of group differences in primary MIND gradient between MS and NMOSD. MIND, Morphometric INverse Divergence; MS, multiple sclerosis; NMOSD, neuromyelitis optica spectrum disorder.
**Supplementary Figure 5**. Spatial cross‐validation of PLS‐derived transcriptomic‐gradient associations. Twenty iterations of leave‐25%‐out spatial resampling were performed in MS and NMOSD, respectively. The y‐axis represents Spearman correlation coefficients. MS, multiple sclerosis; NMOSD, neuromyelitis optica spectrum disorder.

**Supplementary Table 1.** Regional values of the primary MIND gradient.
**Supplementary Table 2**. Case–control comparisons of MIND gradient between MS patients and healthy controls.
**Supplementary Table 3**. Case–control comparisons of MIND gradient between NMOSD patients and healthy controls.
**Supplementary Table 4**. Regional differences in the primary MIND gradient between MS and NMOSD.
**Supplementary Table 5**. The significant gene list of PLS2 genes in MS.
**Supplementary Table 6**. The significant gene list of PLS2 genes in NMOSD.
**Supplementary Table 7**. Functional annotations for PLS2 genes in MS and NMOSD.
**Supplementary Table 8**. Cellular enrichment analysis for PLS2 genes in MS and NMOSD group.
**Supplementary Table 9**. Temporal enrichment analysis of PLS2 genes in MS and NMOSD within cortical regions.

## Data Availability

The raw data that support the findings of this study are available upon reasonable request from the corresponding author.
