# Supplementary material for: Cortical Hierarchical Disorganization and Transcriptomic Associations in Multiple Sclerosis and Neuromyelitis Optica Spectrum Disorders Revealed by Morphometric INverse Divergence Network Gradients
Source: CNS Neurosci Ther. 2026 Nov 9;32(11):e71240. doi: 10.1002/cns.71240 (PMC13748816; doi:10.1002/cns.71240)
Supplement: Supplementary file 1 [file CNS-32-e71240-s001.docx]

**Supplementary Figure 1.** Variance explained by the first ten gradient components.

**Supplementary Figure 2. Correlations between gradient values obtained at 10% sparsity and those obtained at 5% and 15% sparsity.** Abbreviations: MS, multiple sclerosis; NMOSD, neuromyelitis optica spectrum disorder; HC, healthy controls.

**Supplementary Figure 3. Cortical regions showing concordant and divergent patterns of MIND-gradient alterations between MS and NMOSD.** Abbreviations: MS, multiple sclerosis; NMOSD, neuromyelitis optica spectrum disorder.

**Supplementary Figure 4. Distribution of group differences in primary MIND gradient between MS and NMOSD.** Abbreviations: MIND, Morphometric INverse Divergence; MS, multiple sclerosis; NMOSD, neuromyelitis optica spectrum disorder.

**Supplementary Figure 5. Spatial cross-validation of PLS-derived transcriptomic-gradient associations.** Twenty iterations of leave-25%-out spatial resampling were performed in MS and NMOSD, respectively. The y-axis represents Spearman correlation coefficients. Abbreviations: MS, multiple sclerosis; NMOSD, neuromyelitis optica spectrum disorder.
